# Combustion-derived particles from biomass sources differently promote epithelial-to-mesenchymal transition on A549 cells

**DOI:** 10.1007/s00204-021-02983-8

**Published:** 2021-01-22

**Authors:** Sara Marchetti, Rossella Bengalli, Pamela Floris, Anita Colombo, Paride Mantecca

**Affiliations:** POLARIS Research Centre, Department of Earth and Environmental Sciences, University of Milano-Bicocca, Piazza della Scienza 1, 20126 Milan, Italy

**Keywords:** Biomass combustion-derived particles, Epithelial-to-mesenchymal transition, Interleukin-8, Lung cancer

## Abstract

Combustion-derived particles (CDPs), due to the presence in their composition of several toxic and carcinogenic chemical compounds, such as polycyclic aromatic hydrocarbons (PAHs) and metals, are linked to several respiratory diseases, including lung cancer. Epithelial-to-mesenchymal transition (EMT) is a crucial step in lung cancer progression, involving several morphological and phenotypical changes. The study aims to investigate how exposure to CDPs from different biomass sources might be involved in cancer development, focusing mainly on the effects linked to EMT and invasion on human A549 lung cells. Biomass combustion-derived particles (BCDPs) were collected from a stove fuelled with pellet, charcoal or wood, respectively. A time course and dose response evaluation on cell viability and pro-inflammatory response was performed to select the optimal conditions for EMT-related studies. A significant release of IL-8 was found after 72 h of exposure to 2.5 μg/cm^2^ BCDPs. The EMT activation was then examined by evaluating the expression of some typical markers, such as E-cadherin and N-cadherin, and the possible enhanced migration and invasiveness. Sub-acute exposure revealed that BCDPs differentially modulated cell viability, migration and invasion, as well as the expression of proteins linked to EMT. Results showed a reduction in the epithelial marker E-cadherin and a parallel increase in the mesenchymal markers N-cadherin, mainly after exposure to charcoal and wood. Migration and invasion were also increased. In conclusion, our results suggest that BCDPs with a higher content of organic compounds (e.g. PAHs) in their chemical composition might play a crucial role in inducing pro-carcinogenic effects on epithelial cells.

**Supplementary Information:**

The online version contains supplementary material available at 10.1007/s00204-021-02983-8.

## Introduction

International Agency for Research on Cancer (IARC) has categorized biomass burning as probably carcinogenic for humans (Group 2a) (IARC 2010a). Biomass combustion is indeed accountable of the emission in the atmosphere of several toxic and carcinogenic chemical substances at variable concentrations, including polycyclic aromatic hydrocarbons (PAHs) and metals (Duan et al. 2009; IARC 2010a). In recent years, numerous PAHs have been documented as carcinogenic compounds group 1 and 2. Some specific compounds have been indeed reported in literature to be carcinogenic and mutagenic, such as phenanthrene, fluoranthene, pyrene, chrysene, benzo(a)anthracene, benzo(b)fluoranthene, benzo(j)fluoranthene, benzo(k)fluoranthene, benzo(e)pyrene, benzo(a)pyrene, indeno(1,2,3,c,d)pyrene and dibenzo(a,h) anthracene (IARC 2013; IARC 2010b; Sarigiannis et al. 2015). Although PAHs have been positively associated to various genotoxic and carcinogenic effects, transition and heavy metals are also reported as contributor for adverse health effects (IARC 2010a, b; Uski et al., 2015; Van Den Heuvel et al. 2016; Yang et al. 2017).

Biomass combustion-derived particles (BCDPs), together with diesel exhaust particles (DEP) derived from traffic, are the major sources of particulate matter (PM) and ultrafine particles (UFPs) emitted in the urban areas (Corsini et al. 2019). Therefore, long-term exposure to such BCDPs may concur in cancer development associated to air pollution (Fullerton et al. 2008). According to the World Health Organization (WHO), ambient air pollution is responsible for an estimated 4.2 million deaths per year due to cardiovascular diseases, including stroke, and respiratory pathologies such as chronic obstructive pulmonary disease (COPD) and lung cancer. Approximately, 6% of lung cancer deaths are attributable to PM globally (WHO 2014). As a multifactor-causing disease and a long-term process, cancer development is also a result of exposure to environmental pollutants, including biomass combustion products (Bersaas et al. 2016; Kim et al. 2011).

Epithelial–mesenchymal transition (EMT) is a relevant process associated with embryogenesis, organ development, wound healing and tissue repair. However, it plays also a harmful role in cell adhesion, chronic inflammation diseases (such as fibrosis) and tumor migration, invasion and metastasis (Bersaas et al. 2016; Chen et al. 2016). EMT may occur in different epithelial tumors, including lung cancer (Liu et al. 2013).

During EMT, cancer cells of epithelial origin go through multiple biochemical changes, losing cellular polarity and adhesion contacts (Yang et al. 2017; Zou et al. 2013) and re-organizing the cytoskeleton (Liu et al. 2013). Finally, cells differentiate into a mesenchymal-cell phenotype, which is described by improved motility, invasiveness and resistance to apoptosis (Kalluri and Weinberg, 2009). Several genes have been reported as key regulators for cancer progression. Indeed, EMT, is characterized by loss of epithelial markers, like E-cadherin and ZO-1, and gain of mesenchymal-type proteins, such as Vimentin and N-cadherin (Bersaas et al. 2016; Thiery et al. 2009).

Furthermore, soluble factors, including the tumor growth factor beta (TGF-β) and the interleukin-8 (IL-8), could contribute to promote and/or maintain an EMT phenotype (Palena et al. 2012; Xiao and He 2010). The increased release of cytokines and chemokines is a common response after PM exposure (Sun 2010; Van Eeden et al. 2001) and chronic inflammation due to airborne particles could lead to lung remodeling (Ohbayashi 2002).

Biomasses represent widely diffuse fuels for domestic heating. However, to our knowledge, only Longhin et al. (2016) have investigated their potential carcinogenic risk focusing on EMT as crucial pathogenetic mechanism. Nevertheless, studies have been performed on cigarette smoke-induced EMT (Bersaas et al. 2016; Milara et al. 2013; Shen et al. 2014).

We have previously shown the key role of the chemical composition of particles emitted from the same combustion technology, using different biomasses, on several toxicological responses in human lung epithelial cells (A549) (Marchetti et al. 2019). In the present study, we assessed sub-acute toxicity and EMT process activation induced by different BCDPs on A549 cells. Indeed, several studies have already reported the ability of A549 to undergo EMT in vitro after PM, cigarette smoke condensate or transforming growth factor-β1 (TGF-β1) exposure, corroborating their use for carcinogenesis investigations (Kasai et al. 2005; Morales-Bárcenas et al. 2015; Yang et al. 2017; Zou et al. 2013). Among these, Buckley et al. (2010) described the ability of different epithelial cells, including lung cells, to undergo EMT when exposed to the TGF-β1 and other pro-inflammatory mediators, showing that only A549 cells displayed an EMT-like phenotype, compared to the other investigated cell lines.

Our aim was to identify the possible mechanisms by which BCDPs may exert their pro-carcinogenic properties and thus increase the risk of cancer. Attention was given to EMT activation and the consequent increase in motility and invasiveness, also taking into account the BCDP content of PAHs and metals. The present study points out the importance of selecting biomass fuels as energy source considering both composition and biological activity, in order to reduce lung cancer risk.

## Materials and methods

### BCDP sampling and extraction

Samplings were performed using an open fireplace fuelled for 4 h with pellet, charcoal or wood, respectively. Briefly, BCDPs were collected on Teflon filters using a gravimetric sampler. Filters from the same fuel were pooled and particles mechanically detached using an ultrasound bath (Sonica Soltec, Milan, Italy). The obtained CDPs water suspensions were then aliquoted, dried and stored until use. Finally, particles were resuspended in sterilized water to obtain suspensions at a final concentration of 2 µg/µL and sonicated just prior to cell exposure. Particles showed different chemical analysis, with charcoal and wood ones having rather similar composition, characterized by a consistent amount of PAHs (about 10 and eightfold higher than pellet). Pellet instead, resulted to be enriched in metals.

More details on BCDPs sampling, extraction and physico-chemical characterization have been reported in our previous study (Marchetti et al. 2019).

### Cell culture

Human alveolar epithelial cell line A549 was purchased from the American Type Culture Collection (ATCC® CCL-185, American Type Culture Collection, Manassas, USA). Cells were cultured in complete medium at 37 °C in humidified atmosphere with 5% CO_2_. Complete culture medium was composed of Optimem medium supplemented with 10% heat-inactivated Fetal Bovine Serum (FBS) and penicillin/streptomycin (0.1 mg/mL). For experiments, cells were seeded and grown up for 24 h in complete medium. The day after, the culture medium was replaced with Optimem medium with 1% FBS (exposure media) and particle suspension added directly to it. Cells were exposed also to 7 µM benzo[a]pyrene (B[a]P, Sigma Aldrich, Saint Louis, MO, USA) for xenobiotic metabolism activation studies or 5 ng/mL TGF-β (Sigma) for EMT experiments (protein expression, migration, invasion), as positive control (see Supplemental file 1). All the in vitro experiments were performed at least in triplicate.

### Cell viability

A549 viability was assessed by means of Alamar Blue (Life Technologies, Monza, Italy) assay accordingly to manufacturer’s instruction, as previously described (Marchetti et al. 2019). Depending on the incubation time, A549 cells were seeded at different density (2 × 10^4^ cell/cm^2^ for 24 h of exposure, 1 × 10^4^ cell/cm^2^ for 48 h and 5 × 10^3^ cell/cm^2^ for 72 h) and exposed to the different BCDPs after 24 h. For each source, cells were exposed to different doses (1, 2.5 and 5 µg/cm^2^) for 24, 48 and 72 h. Then, a solution containing 1:10 of Alamar Blue reagent and cell complete culture medium was added into each well. After 3 h of incubation, media absorbance was read to the spectrophotometer (TECAN Infinite Pro) at 570 and 630 nm wavelengths and compared to control values.

### ELISA

Supernatants from A549 cells were collected after exposure to BCDPs, centrifuged to remove particles (12,000 rpm, 6 min, 4 °C) and stored at − 80 °C until analysis. IL-8 protein levels were detected after 48 and 72 h of exposure by sandwich ELISA according to the manufacturer’s instructions (Life Technologies). Absorbance was measured by Multiplate Reader Ascent (Thermo Scientific, USA) at the wavelengths of 450 and 650 nm and the amount of proteins expressed in pg/mL based on a standard curve.

### BPDE DNA bulky adducts detection

After a 72 h of exposure to 2.5 µg/cm^2^, DNA was extracted from A549 cell pellets using the FlexiGene DNA kit (Qiagen) and quantified by spectrophotometry. DNA adduct detection was performed using the OxiSelect BPDE DNA Adduct ELISA Kit (Cell Biolabs, San Diego, USA), an enzyme immunoassay that allow detection of benzo(a)pyrene diol epoxide (BPDE)-DNA adducts. Briefly, extracted DNA was diluted to 4 μg/mL in TE Buffer (10 mM Tris, pH 8.0, 1 mM EDTA). DNA samples and BPDE-DNA standards were then added in duplicate to the well of the plate and incubated overnight (O/N) at room temperature (RT) with DNA Binding Solution. The day after, samples were rinsed with PBS and incubated with assay diluent for 1 h at RT. Then, after washing, Anti-BPDE-I Antibody was added for 1 h. Finally, samples were incubated with Secondary Antibody-HRP conjugate for 1 h, and then substrate and stop solution to develop the colorimetric enzyme reaction. Absorbance was read at 450 nm and the amount of BPDE DNA Adduct expressed as fold change compared to the control cells.

### Protein expression analysis

For protein expression evaluation, 4 × 10^3^ cells/cm^2^ were exposed for 72 h to 2.5 μg/cm^2^ BCDPs in 6-well plates. After treatment, cells were lysed on ice with RIPA buffer (150 mM NaCl, 1% Triton X-100, 0.5% sodium deoxycholate, 0.1% SDS, 50 mM Tris pH 8.0) and 0.1% of proteases inhibitor, added just before use. Lysates were then, centrifuged at 12,000 rpm for 15 min to remove debris and particles. Finally, protein concentration was assessed by bicinchoninic acid assay (Sigma Aldrich), according to the manufacturer instructions. Thirty µg of proteins were loaded onto 10% SDS-PAGE gels, separated and transferred on nitrocellulose membranes. Membranes were incubated for 1 h with blocking buffer, composed of Tris-Buffered Saline (TBS) with 0.1% Tween20 (TBS-T) supplemented with 5% w/v bovine serum albumin (BSA; Sigma) or milk (Skim milk powder, Fluka, Sigma). Afterward, membranes were incubated O/N at 4 °C with the following rabbit monoclonal antibodies: ZO-1, E-cadherin, N-cadherin, Vimentin, (1:1000, Cell Signaling Technology, Danvers, USA), CYP1A1 and CYP1B1 (1:500, Novus Biologicals, Littleton, CO, United States). The following day, membranes were washed three times with TBS-T and then incubated with the specific HRP-linked secondary antibodies (anti-rabbit IgG, 1:2000, Cell Signaling) in Blocking buffer for 1 h at RT. Finally, after washing, proteins were detected by enhanced chemiluminescent (ECL, Euroclone) and digital images taken by means of a luminescence reader (Biospectrum-UVP, LLC, Upland, CA, United States). The dedicated software (Vision Works LS) was used to perform densitometry analysis. As loading control, monoclonal anti-β-Actin antibody (Cell Signaling, 1:1000) was used.

### Immunofluorescence microscopy

The analysis of the expression of E-cadherin and N-cadherin, as epithelial and mesenchymal markers respectively, was evaluated also through immunofluorescence analysis. Cells (4 × 10^3^ cells/cm^2^) were seeded on a six-multiwell having coverslides on the bottom. After 24 h of incubation, cells were treated with the different BCDPs (2.5 μg/cm^2^) for 72 h. After treatment, cells were washed twice with PBS and then fixed with 4% paraformaldehyde for 20 min. For staining, cells were washed with PBS and permeabilized with PBTS (1X PBS + 0.3% Triton X-100 and 5% BSA) for 30 min. After permeabilization, cells were stained with the antibody mAb rabbit anti-E-cadherin (24E10) (1:200; Cell Signaling) or anti-N-cadherin (1:200; Cell Signaling) in antibody dilution buffer (1X PBS + 1% BSA + 0.3% Triton X-100) O/N at 4 °C. The secondary antibody goat anti-rabbit Alexa Fluor488 (Molecular Probe, Life Technologies, Monza, Milano, Italy) was added for 2 h at RT after washes. Cells stained with E-cadherin were also stained for 20 min with rhodamine phalloidin (Cytoskeleton Inc., Denver, CO, US) for the evaluation of actin changes. Cells were finally counterstained with DAPI (Molecular Probe, Life Technologies, Monza, Italy) for 2 min and after washing, mounted with ProLong™ Gold Antifade Mountant (Life Technologies) on glass slides. The analysis of fluorescence was evaluated through an AxioObserver Z1 Cell Imaging station (Carl-ZEISS Spa, Milano, Italy) and images were elaborated with the dedicated software ZEN 2.3 Blue edition.

### Migration and invasion assays

To assess cell motility and invasiveness, 1.9 × 10^5^ cells/cm^2^ A549 cells were seeded on polycarbonate membrane inserts with 8 µm pore size. CytoSelect 24—Well Cell Migration and CytoSelect 24-Well Cell Invasion assays were performed according the manufacturer’s manuals (Cell BioLabs). Briefly, cell suspensions were placed in upper chamber of inserts in Optimem medium without serum for O/N starvation. The day after, supernatants in the upper chamber were replaced with exposure media and cells were treated with 2.5 µg/cm^2^ BCDPs for 72 h. Complete media was added to the bottom chamber. Migratory/invasiveness cells pass through basement membrane layer to the bottom chamber of the insert. Non-migratory/invasiveness cells (upper chamber) were removed and inserts incubated with cell stain solution for 10 min at RT. After that, cells were washed several times with sterile water and allow to dry. Finally, extraction solution was added for 10 min incubation. Absorbance was read with a spectrophotometer at 560 nm and migration/invasion of exposed cells expressed as fold change compared to the control cells.

### Statistical analysis

The data represent mean and standard error of mean (SEM) of three independent experiments carried out at the same experimental conditions. Statistical analysis was performed with GraphPad Prism 6 software, using One-way or Two-way ANOVA with Tukey’s, Dunn’s or Dunnett’s post hoc multiple comparisons tests. Values of *p* < 0.05 were considered statistically significant.

## Results

**Fig. 1:**
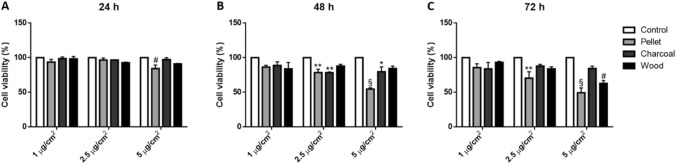
Cell viability. A549 viability was assessed after 24 (**a**), 48 (**b**) and 72 h (**c**) of exposure to increasing doses of BCDPs (1, 2.5 and 5 µg/cm^2^). Histograms represent cell viability expressed as percentage compared to the control (expressed as 100%). Each bar shows mean ± SEM of three independent experiments (*N* = 3). Statistical analysis was performed by Two-way ANOVA with Tukey’s multiple comparisons test. ^§^*p* < 0.0001, ^#^*p* < 0.001, **p < 0.01 and *p < 0.05 vs. control cells

Viability assay showed that pellet induced a dose response mortality, with effects visible already after 24 h of exposure, as previously reported (Marchetti et al. 2019). The cytotoxic response is increased after 48 and 72 h, showing 2.5 µg/cm^2^ as sub-lethal dose (Fig. 1). Charcoal instead did not induce significant effects on cell viability except for a slight reduction after long-term exposure (Fig. 1b, c). Regarding wood, a decreased viability was observed after 72 h of exposure to 5 µg/cm^2^ (Fig. 1c).

**Fig. 2:**
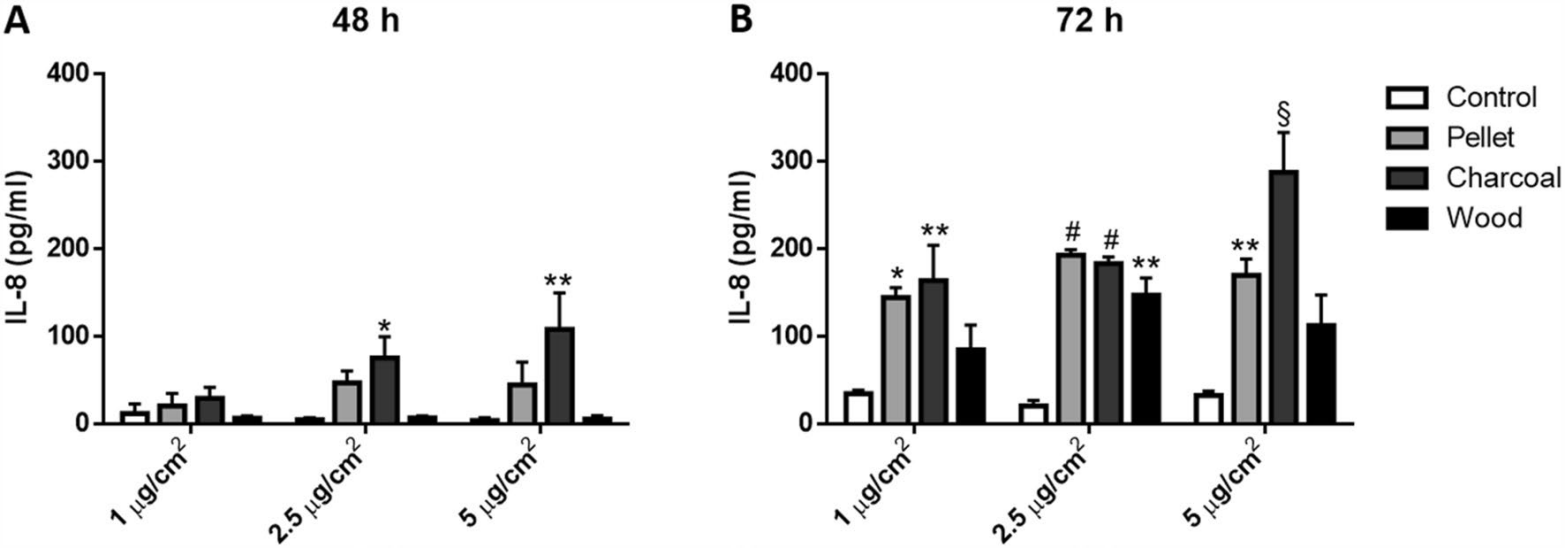
Pro-inflammatory response. IL-8 protein secretion after 48 (**a**) and 72 h (**b**) of A549 exposure to increasing BCDPs doses (1, 2.5 and 5 µg/cm^2^). Each bar shows mean ± SEM of three independent experiments (*N* = 3). Statistical analysis was performed by Two-way ANOVA with Tukey’s multiple comparisons test. §*p* < 0.0001, #*p* < 0.001, **p < 0.01 and *p < 0.05 vs control cells

As a measure of BCDPs-induced pro-inflammatory effects, and as possible soluble factor involved in promoting EMT, the release of IL-8 was detected after 48 h and 72 h of exposure (Fig. 2). Although the activation of the inflammatory response was not detected after 24 h of exposure to BCDPs, IL-8 secretion was discovered augmented both at 48 and at 72 h of exposure. Indeed, results showed an increased release in time of the cytokine with all BCDPs and doses tested. Charcoal and pellet seemed the most potent BCDPs, inducing IL-8 release at 2.5 µg/cm^2^ 9 and 8 times more than control, respectively.

Considering the results obtained on cell viability and IL-8 release, 72 h of exposure and the dose of 2.5 µg/cm^2^ were selected to perform the following experiments.

**Fig. 3:**
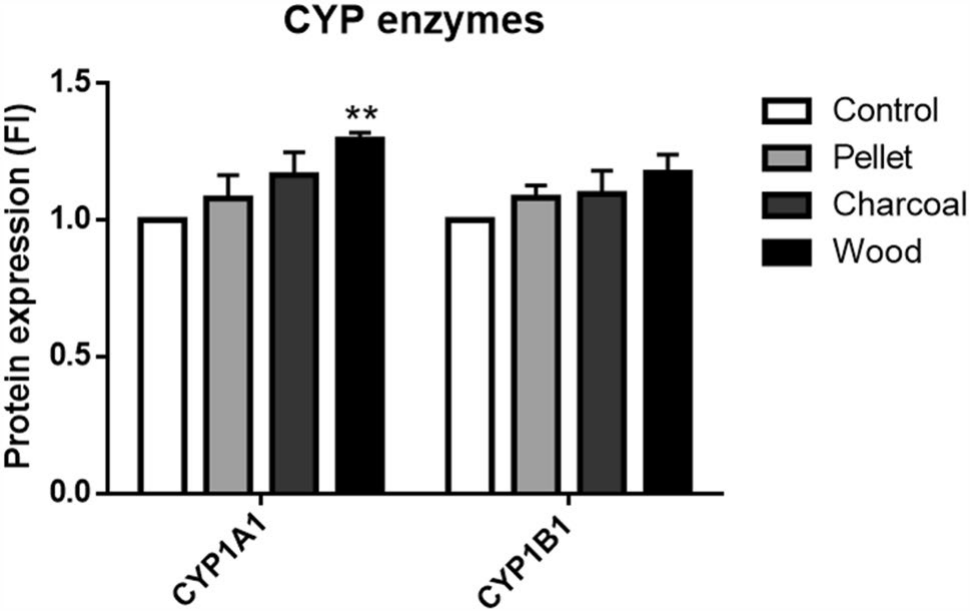
CYP1A1 and CYP1B1 protein expression in A549 cells after 72 h of exposure to 2.5 µg/cm^2^. Each bar shows mean ± SEM of three independent experiments (*N* = 3). Statistical analysis was performed by One-way ANOVA with Sidak's multiple comparisons test. **p < 0.01 vs. control cells

The metabolic activation of PAHs adsorbed on BCDPs and PAH-induced DNA bulky adduct formation were then studied. A statistically significant increase in CYP1A1 protein activity was detected only in cells exposed to wood, while a slight effect was exerted on CYP1B1 by all BCDPs (Fig. 3).

However, no BPDE-DNA bulky adduct were detected, regardless the type of biomass source (Supplementary File 2, Fig. 1).

**Fig. 4:**
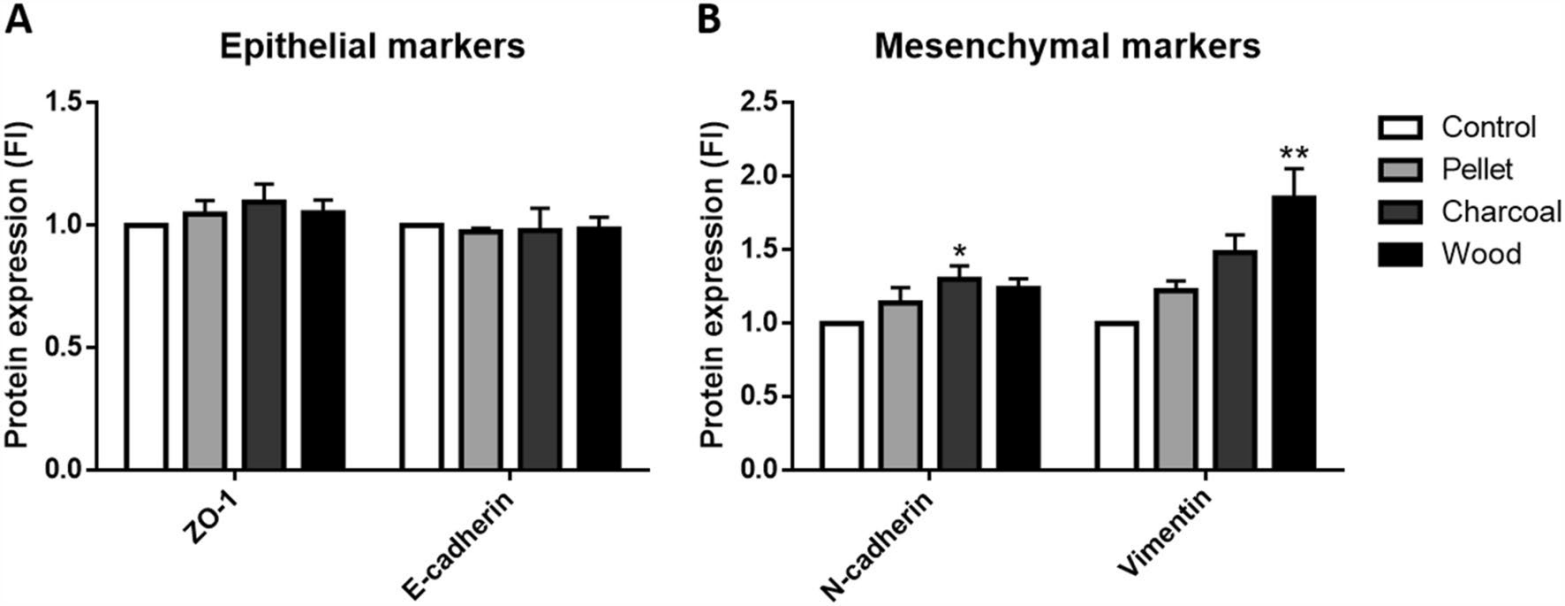
EMT-related proteins expression on A549 cells after 72 h of exposure to 2.5 µg/cm^2^. **a** Epithelial-type proteins: ZO-1 and E-cadherin. **b** Mesenchymal-type proteins: N-cadherin and Vimentin. Each bar shows mean ± SEM of three independent experiments (*N* = 3). Statistical analysis was performed by One-way ANOVA with Dunnett’s or Dunn’s multiple comparisons test. **p < 0.01 and *p < 0.05 vs. control cells

EMT-proteins expression on A549 cells was also evaluated in order to verify if BCDPs might modulate the process activation. After exposure to BCDPs, the levels of expression of epithelial markers were not influenced (Fig. 4a). However, mesenchymal markers (Fig. 4b) were found modulated. Charcoal significantly increased both N-cadherin and modulated Vimentin, although not significantly. Wood instead, significantly increased only the expression of Vimentin. No significant effects were detected after exposure to pellet.

**Fig. 5:**
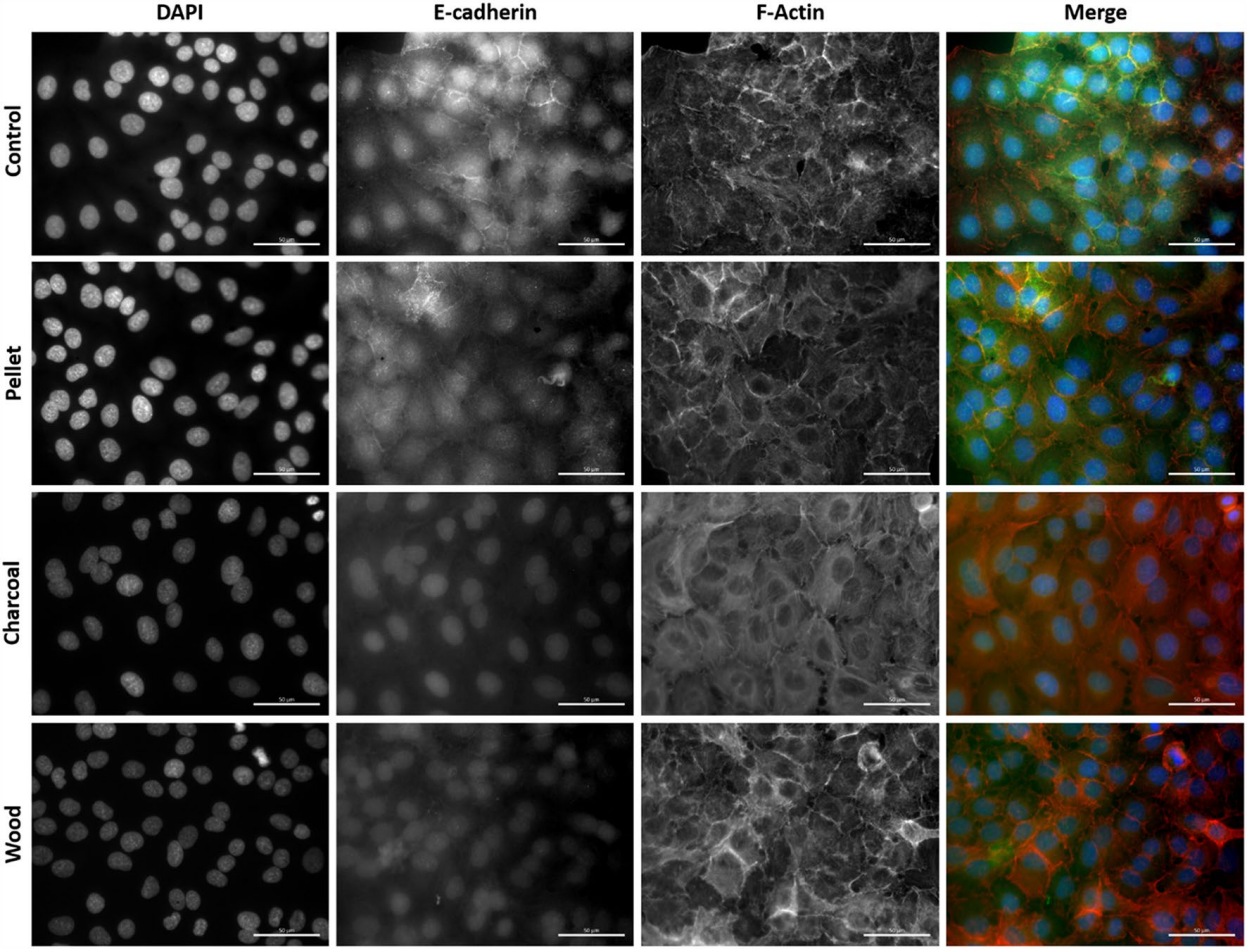
Immunofluorescence of the EMT epithelial marker E-cadherin on A549 cells after 72 h of exposure to BCDPs (2.5 µg/cm^2^). Nuclei are stained with DAPI (blue); E-cadherin with rabbit anti-E-cadherin (green) antibody and F-actin with rhodamine-phalloidin (red). Scale bar = 50 µm (color figure online)

**Fig. 6:**
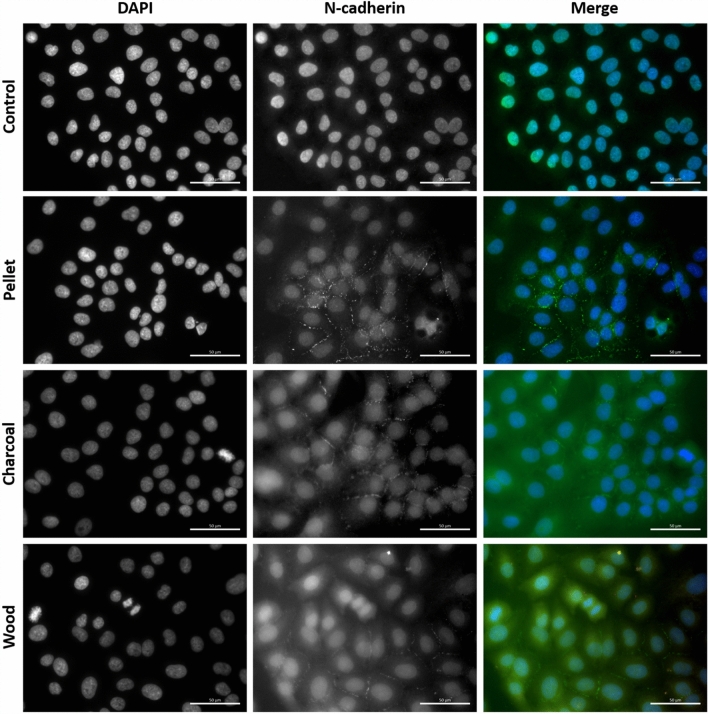
Immunofluorescence of the EMT mesenchymal marker N-cadherin on A549 cells after 72 h of exposure to BCDPs (2.5 µg/cm^2^). Nuclei are stained with DAPI (blue) and N-cadherin with anti-body rabbit anti-N-cadherin (green). Scale bar = 50 µm (color figure online)

The expression of epithelial and mesenchymal markers in BCDPs exposed cells was detected also by immunostaining (Figs. 5 and 6). Here, we observed that in the exposed cells, especially charcoal and wood, E-cadherin is expressed mainly in the cytoplasm, and not in the plasma membrane. This delocalization after exposure to BCDPs suggests a reduction in the cell-to-cell contacts.

A cytoskeletal remodeling was also observed with augmented expression of stress fibers, actin ruffles and focal adhesion complexes, as evidenced by the actin staining (Fig. 5 and Supplementary File 2, Fig. 2).

In parallel, N-cadherin, the mesenchymal marker, was found increased upon incubation with BCDPs (Fig. 6), mainly after charcoal and wood exposure, confirming western blot analysis.

**Fig. 7:**
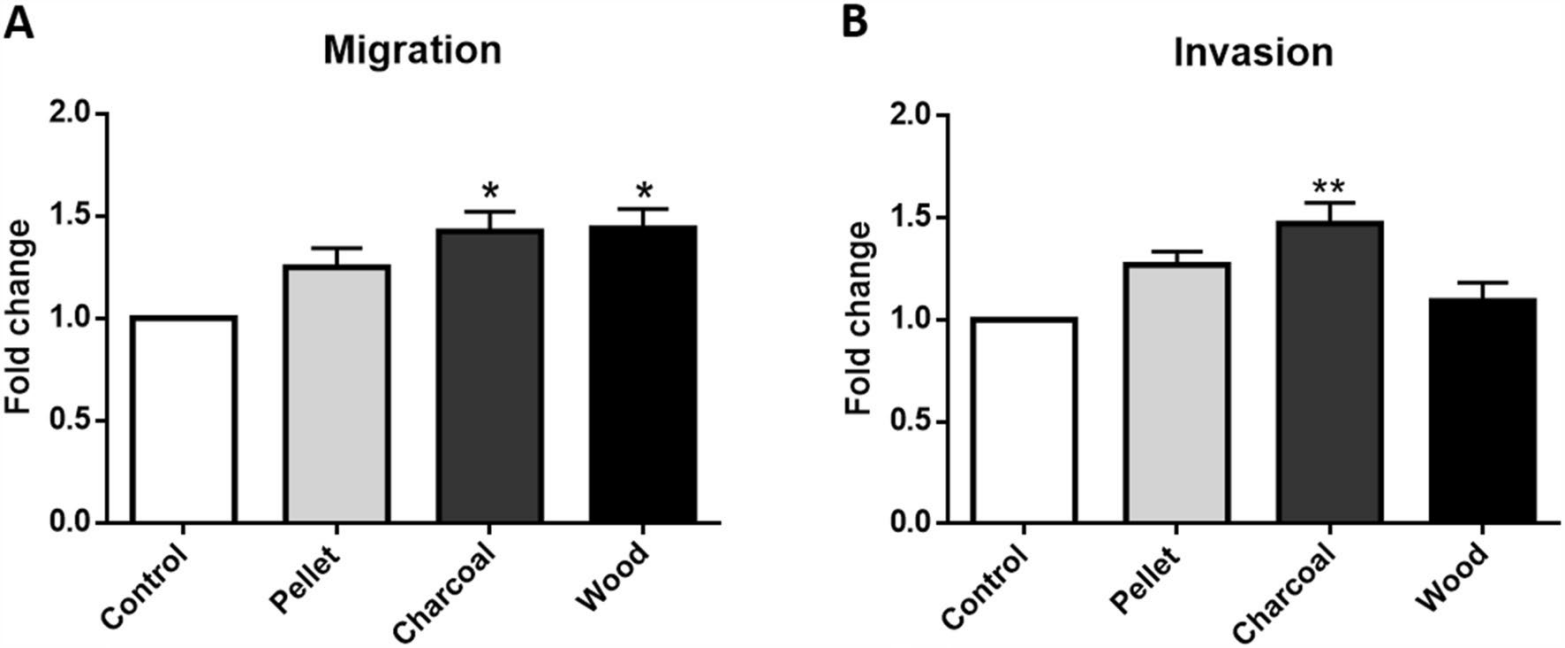
Effects of BCDPs on A549 migration (**a**) and invasion (**b**) after 72 h of exposure to BCDPs (2.5 µg/cm^2^). Each bar shows mean ± SEM of three independent experiments (*N* = 3). Statistical analysis was performed by One-way ANOVA with Dunnett’s multiple comparisons test. **p < 0.01 and *p < 0.05 vs. control cells

Transwell assays were then performed to assess if BCDPs could stimulate A549 migration and invasiveness activity (Fig. 7). As shown in Fig. 7a, migration of charcoal and wood-exposed cells was significantly enhanced compared to the control. Furthermore, we conducted a transwell invasion assay. Results showed that A549 cells exposed to charcoal significantly enhanced their ability to invade (Fig. 7b), degrading the basement membrane matrix proteins in the layer, and ultimately passing through the pores of the polycarbonate membrane. No effects on invasion were found instead after exposure to wood. About pellet, only slight differences in migration and invasion were detected compared to the control. Taken together, the results suggested that charcoal was the most effective in promoting A549 cell migration and invasion in vitro.

## Discussion

The dramatic impact of air pollution, especially of airborne PM and UFP, on human health has already been scientifically demonstrated (WHO 2014) and many evidences on the effects of these compounds in the development of lung cancer have been given (Hamra et al. 2014; Pope et al. 2002; Raaschou-Nielsen et al. 2016). However, there are still little information about the mechanisms that are involved in the carcinogenic process, especially in relation to exposure to airborne particles emitted from different emission sources.

Besides, combustion-derived particles (CDPs), including PM derived from biomass burning (BCDPs), are considered one of the major risk factors for the development of respiratory diseases, including lung cancer (Stabile et al. 2018). This evidence poses a huge concern for worldwide population health safety and implies both ethical and economic issues that must be urgently dealt. Furthermore, the data from in vivo and in vitro air monitoring and clinical studies push not only researchers, but also policy makers and international agencies, to find new strategies for reducing particle emissions in the atmosphere. The use of fuels that produce BCDPs with less impacting chemical composition on human health could be helpful in reducing the morbidity and mortality related to the onset of lung tumors. In this perspective, in vitro studies could be useful tools to understand the key events and the molecular mechanisms of the biological outcomes associated to BCDPs exposure.

In lung cancer, epithelial cells play an important role during lung remodeling, which could lead to progression of respiratory diseases. Since there are much missing information about the specific role played by BCDPs, here, we aimed to investigate their role in some mechanisms known to be involved in lung cancer development. Basing on previous results on the genotoxic effects of biomass PM in A549 cells (Marchetti et al. 2019), this alveolar epithelial cell line has been used to further contribution of BCDPs in the progression of the carcinogenic events. Although of carcinogenic origin, A549 cell line is in fact a widely and currently accepted experimental model for in vitro inhalation toxicology studies. Furthermore, A549 cells maintain alveolar type II cells characteristics, such as secretion of cytokines, surfactant production and phase I and phase II enzymes for xenobiotic biotransformation similar to lung tissue (Deng et al. 2014; Orona et al. 2014).

The A549 cells were exposed to sub-toxic doses of PM derived from different biomass sources, such as pellet, charcoal and wood, having different chemical composition. Effects of BCDPs on A549 cell viability, inflammatory response, xenobiotic enzymes activation, DNA bulky adducts formation, expression of epithelial and mesenchymal protein markers, migration and invasion were investigated. Previous works have demonstrated that exposure to PM10 and PM2.5 modulates the expression of different genes involved in xenobiotic transformation, inflammation and EMT pathways and that the up-regulation or down-regulation of these genes is related to the variable chemical composition of PM sampled in different seasons (Gualtieri et al. 2012; Yue et al. 2015) or derived from different emission sources (Longhin et al. 2016).

BCDPs contains several chemical compounds, both organic and inorganic, including PAHs. Among these, some PAHs have been reported indeed to be carcinogenic and largely contribute to cancer risks, including benzo[a]pyrene (B[a]P). In recent years, increasing evidence have described the crucial role of PAHs in lung carcinogenesis (Yue et al. 2015; Zhang et al. 2016). Here, we analyzed the role of PAHs present in the BCDPs mixtures from pellet, charcoal and wood, considering their genotoxic properties and particularly their possible involvement in EMT activation. Since DNA damage and mutation appearance mostly explained PAHs carcinogenic properties, we analyzed first the possible formation of DNA adduct, reported to be their main genotoxic effect (Genies et al. 2016).

We have previously observed that at early time points (24 h) DNA damage occurs only after pellet exposure, which is the CDP with the most abundant content of metals, especially Zn^+^, compared to the other biomasses (Marchetti et al. 2019).

At later time points, 48 and 72 h, it seems that cells respond also to BCDPs with higher PAHs content, as evidenced by the significant increase in CYP1A1 expression after 72 h of exposure to wood particles (Fig. 3). A reduced effect was instead detected after exposure to pellet particles that had no significant effect on the mechanisms investigated. The effect of PAHs-induced biological responses at 24 h of exposure could be at lower extent because of the latency for the induction of the enzymatic system and as a matter of the fact that PAHs are adsorbed onto carbonaceous particles, which may delay their bioavailability to the CYP enzymatic complex. On the counterpart, metals adsorbed onto PM, such as Zn^+^, can solubilize in the cell culture medium or once internalized in the endo-lysosomal system and exert their effect (e.g. DNA damage) at earlier time points as previously observed (Marchetti et al. 2019).

After CYPs expression evaluation, we measured the formation of DNA adducts of benzo[a]pyrene diol epoxide (BPDE), which has been reported to be strictly connected to CYP1A1-metablized PAHs (Orona et al. 2014; Yue et al. 2015). However, DNA adducts were not detected, confirming that PAHs, when in a complex mixture, interact with each other in several ways in terms of toxicity, potentiating or inhibiting their action, as suggested by Tarantini and Billet (Billet et al. 2018; Tarantini et al. 2011). Although additional evidences are required, the lack of DNA adducts may suggest that a non-genotoxic mechanism could be involved in BCDP-induced carcinogenic events. Epigenetic changes in tumor initiation and promotion after PM and/or PAH exposure have been indeed recently described in literature (Leclercq et al. 2017; Li et al. 2017; Soberanes et al. 2012).

Lung cancer results from a variety of interactions and biological processes, including activation of a strong inflammatory response, vessel development, cell migration and proliferation and, finally, invasion (Fidler 2002). The imbalance caused by BCDPs exposure to lung cells could contribute to the development of cancer through the cell transformation from epithelial to mesenchymal phenotype. Epithelial-to-mesenchymal transition (EMT) is a phenotypic switch that stimulate the acquirement of fibroblastoid-like characteristics by epithelial cancer cells, resulting in increased cell motility and invasiveness, as well as metastatic predisposition and resistance to therapies (Xiao and He 2010). Cells undergoing EMT are also known to increase the secretion of specific mediators, such as cytokines, which could play an important role in cancer progression and invasion (Yue et al. 2015; Zhang et al. 2016). Invasion is defined as cell movement through a 3D matrix, and it requires migration and proteolysis of extracellular matrix components (Kramer et al. 2013).

In vitro alterations associated with an invasive phenotype induced by PM10, were observed in cells exposed to particles with the higher content of PAHs. Indeed wood and mostly charcoal significantly enhanced cell migration and invasion, as well as increased IL-8 secretion in A549 cells, suggesting that biomass combustion may be a noteworthy contributor in lung cancer development and progression. Our data suggest that the up-regulation of N-cadherin and Vimentin, as well as the loss of E-cadherin and disassembly of actin concur to the migration and invasiveness of lung cells exposed to charcoal and wood BCDPs.

Data from invasion assay are in accordance with the work of Morales-Barcenas (2015), in which the authors found alterations in A549 after exposure to sub-toxic dose of PM10 (10 µg/cm^2^) and a more aggressive in vitro phenotype, with an increase in protease activity and invasiveness. Furthermore, previous data (Yue et al. 2015) have evidenced that higher content of PAHs could promote EMT in A549 cells exposed to PM10 and PM2.5 collected from peri-urban sites in North China. The authors found that the winter PAH-bound PM promote lung cancer and that these particles had the characteristics of coal. BCDPs chemical composition have similarities with PM2.5 sampled during winter (PM2.5w) in Milan, as evidenced by the high PAHs and heavy metals content, while particles sampled in summer usually have a major content of crustal elements and biogenic fraction (Gualtieri et al. 2010; Longhin et al. 2013). Moreover, it has been previously demonstrated (Gualtieri et al. 2012) that PM2.5w and BaP shared the down-regulation of the E-cadherin gene (CDH1) associated to the EMT and the up-regulation of other genes involved in these pathways.

As previously mentioned, hallmarks for EMT include dissolution of cell–cell contacts, production of transcription factors able to inhibit or delocalize Epithelial cadherin (E-cadherin) expression, increased expression of mesenchymal markers (e.g. N-cadherin and Vimentin), induction of focal adhesion turnover and secretion of proteolytic enzymes involved in matrix degradation, such as the matrix metalloproteinases (MMPs). Disruption of the actin cytoskeleton could induce an epithelial transition in metastatic cancer cells (Shankar and Nabi 2015). During EMT, the remodeling of actin cytoskeleton and focal adhesion formation are associated. Focal adhesions link the cytoskeleton to the extracellular matrix (ECM) and allow the cell to respond to its environment. Focal adhesions are also sites of localized signal transduction events that modulate processes such as cell proliferation, differentiation and migration. Changes in cell morphology, such as membrane ruffles and filopodia formation might be a consequence of the cytoskeletal remodeling (Yilmaz and Christofori 2009). Moreover, Arjonen and colleagues (Arjonen et al. 2011) reported that filipodia-inducing genes are involved in cancer progression. They also related the altered expression of integrins to negative prognosis in human cancer.

In this regard, a migrating phenotype resulting in an increase of F-actin fibers (Vallenius 2013) has been here observed in A549 exposed to BCPDs. Sanchez-Perez and colleagues have previously reported that PM10 exposure increases the expression of these fibers in lung cells through the stabilization of p21 (Sánchez-Pérez et al. 2014). This change in actin organization and polymerization promote cells migration, giving them characteristics of cancer cells.

IL-8 is a well-known pro-inflammatory cytokine released after PM exposure (Wu et al. 2018). Nevertheless, it is worthy to mention that the development of an EMT phenotype could also depend on the variety of external soluble factors (e.g. cytokines, growth factors or components of the extracellular matrix), which are provided by the tumor microenvironment (Raman et al. 2007; Zhang et al. 2016). These signals can derive from many cells that are within the tumor stroma (e.g. endothelial cells, immune cell, fibroblasts) and they can be capable to induce EMT in the neighboring cancer cells. Furthermore, cancer cells themselves may reprogram the surrounding tumoral tissue by producing cytokines and other soluble mediators that promote tumor growth, dissemination and invasion and that can induce and/or maintain EMT in tumor cells (Fidler 2002). Well-established signals that promote EMT in various tumor cell models are induced by TGF-β and growth factors including FGF, EGF and HGF (Xiao and He 2010). Zhang et al. (2016) reported that by silencing IL-8 and other inflammatory markers, migration is drastically reduced in A549 cells exposed to B[a]P.

The IL-8/IL-8 receptor (IL-8R) axis has been demonstrating to have a role on the induction and/or maintenance of EMT by an autocrine loop and in the remodeling of the tumor microenvironment (Palena et al. 2012). Data from our work evidenced that there is a significant release of IL-8 from cells exposed to charcoal and wood BCDPs for 48 and 72 h, and consequently a modulation of EMT proteins occurred, as proved by E-cadherin, N-cadherin and Vimentin expression and by the migration and invasion assays data.

## Conclusions

**Fig. 8:**
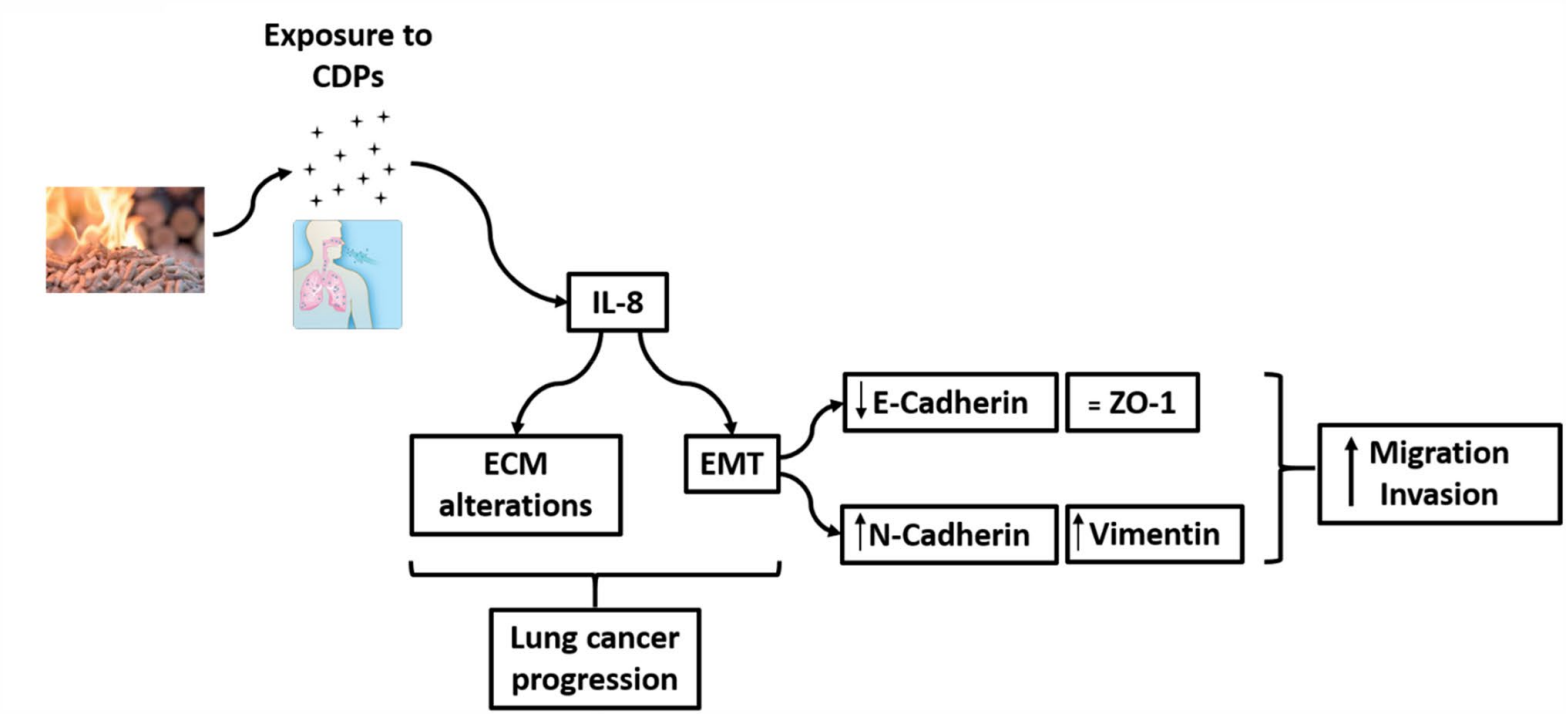
Graphical scheme of the experimental design and the mechanisms involved in the A549 cells response to BCDPs exposure

To conclude, our results support the hypothesis that exposure to BCDPs with high content of PAHs promotes cell migration and invasiveness also by creating a tumor microenvironment in which IL-8 secretion supports the development and/or maintenance of EMT that can be used by cancer cells for invasion (Fig. 8).

Additional studies are required to assess the BCDPs-induced molecular mechanisms responsible for the effects observed at pulmonary level. Future studies should be focused on early carcinogenesis process, using more relevant cell lines to obtain further informative data about how BCDPs might exert their adverse outcome and promote cancer development.

## Supplementary Information

Below is the link to the electronic supplementary material.

Supplementary file1 (DOCX 2410 KB)

Supplementary file2 (DOCX 1978 KB)
