# Supplementary material for: Combustion-derived particles from biomass sources differently promote epithelial-to-mesenchymal transition on A549 cells
Source: Arch Toxicol. 2021 Jan 22;95(4):1379–90. doi: 10.1007/s00204-021-02983-8 (PMC8032642; doi:10.1007/s00204-021-02983-8)
Supplement: Supplementary file 1 — Supplementary file1 (DOCX 2410 KB) [file 204_2021_2983_MOESM1_ESM.docx]

**Supplemental file 1**

**Archives of Toxicology**

**Combustion-derived particles from biomass sources differently promote epithelial to mesenchymal transition on A549 cells**

Sara Marchetti^1*^, Rossella Bengalli^1^, Pamela Floris^1^, Anita Colombo^1^, Paride Mantecca^1^

^1^POLARIS Research Centre, Department of Earth and Environmental Sciences, University of Milano-Bicocca, Piazza della Scienza 1, 20126 Milano, Italy

Sara Marchetti: ^1^POLARIS Research Centre, Department of Earth and Environmental Sciences, University of Milano-Bicocca, Piazza della Scienza 1, 20126 Milano, Italy. s.marchetti16@campus.unimib.it

tel: +390264482928

https://orcid.org/0000-0002-8767-9962

* Corresponding author

**
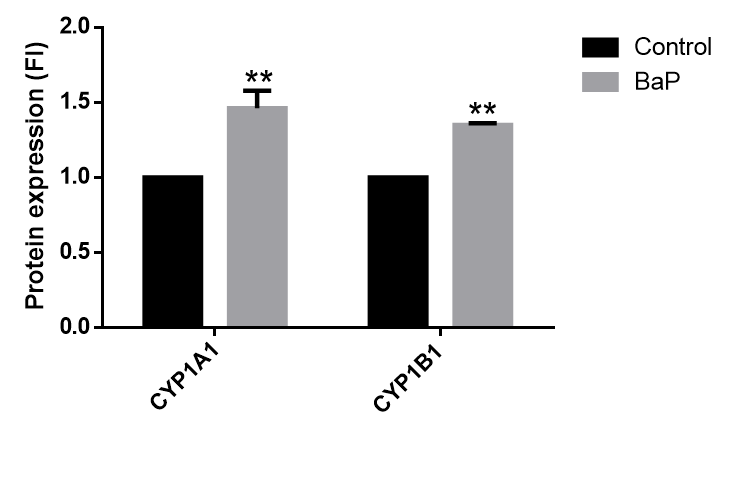
**

**Supplementary Fig. 1** CYP1A1 and CYP1B1 protein expression in A549 cells after 72 h of exposure to B[a]P (7 µM). Each bar shows mean ± SEM of three independent experiments (N=3). Statistical analysis was performed by Two-way ANOVA with Sidak's multiple comparisons test. ***p*<0.01 vs control cells.

**
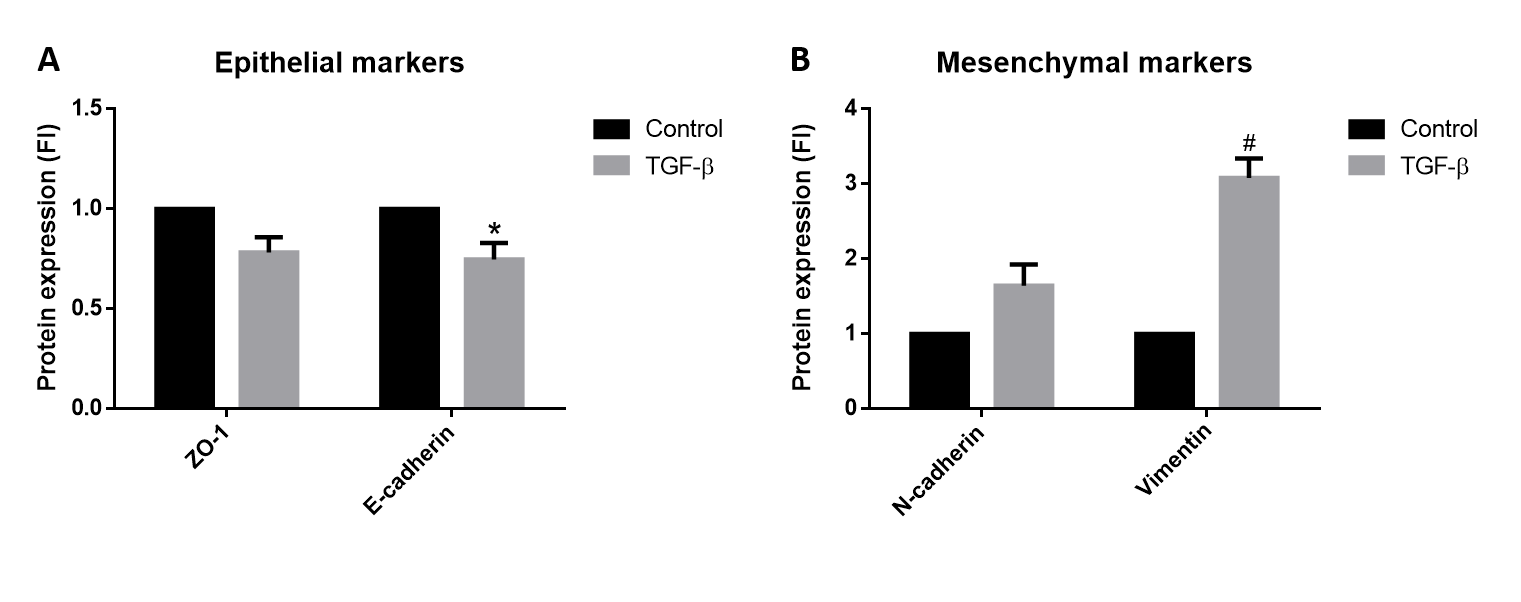
**

**Supplementary Fig. 2** EMT-related proteins expression on A549 cells after 72 h of exposure to TGF-β (5 ng/mL). A) Epithelial-type proteins: ZO-1 and E-cadherin. B) Mesenchymal-type proteins: N-cadherin and Vimentin. Each bar shows mean ± SEM of three independent experiments (N=3). Statistical analysis was performed by Two-way ANOVA with Sidak's multiple comparisons test. ^#^*p*<0.001 and **p*<0.05 vs control cells.


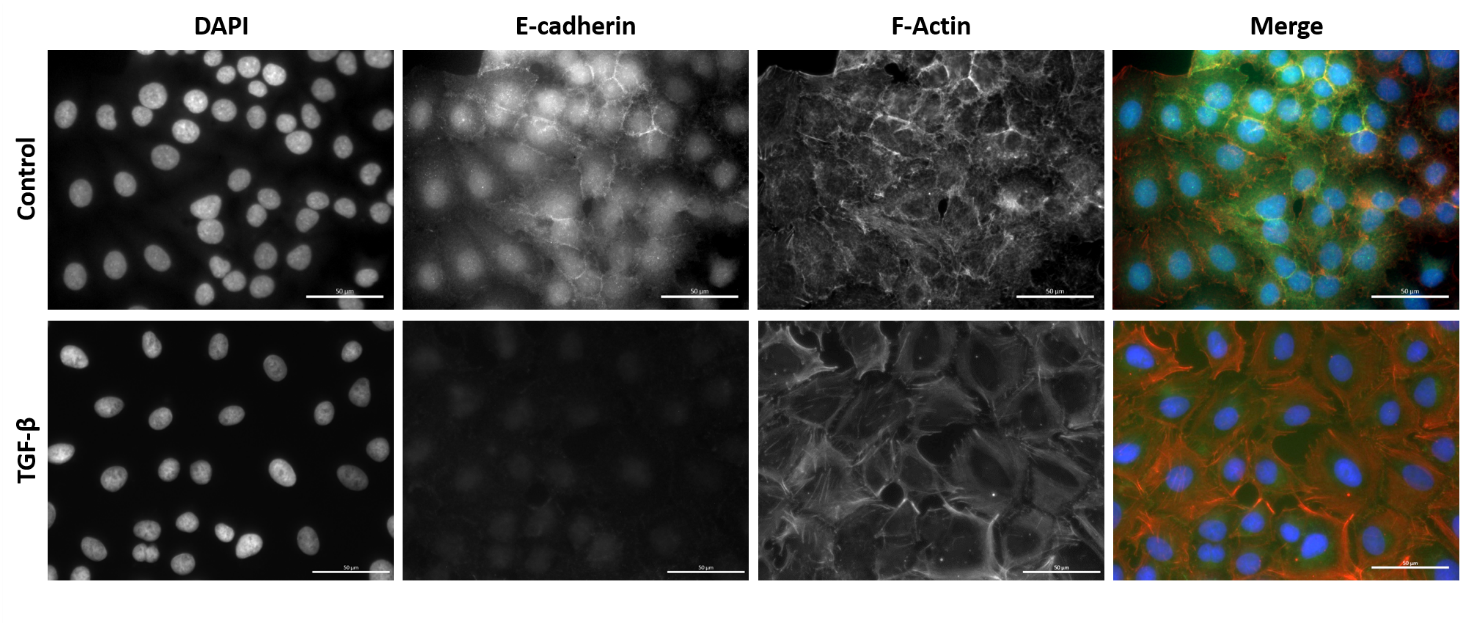
**Supplementary Fig. 3** Immunofluorescence of the EMT epithelial marker E-cadherin on A549 cells after 72 h of exposure to TGF-β (5 ng/mL). Nuclei are stained with DAPI (blue); E-cadherin with rabbit anti-E-cadherin (green) antibody and F-actin with rhodamine-phalloidin (red). Scale bar= 50 µm.

**
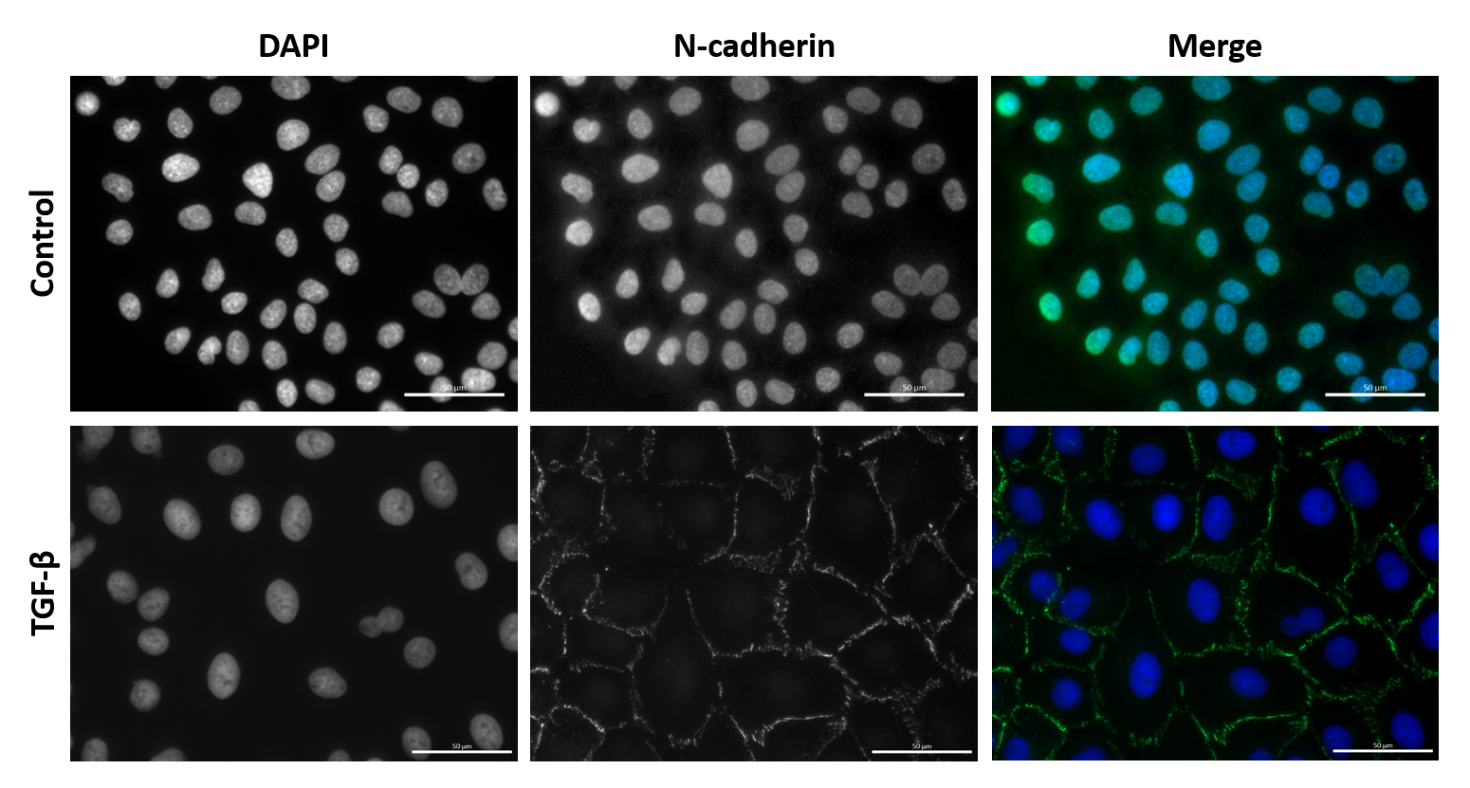
**

**Supplementary Fig. 4** Immunofluorescence of the EMT mesenchymal marker N-cadherin on A549 cells after 72 h of exposure to TGF-β (5 ng/mL). Nuclei are stained with DAPI (blue) and N-cadherin with anti-body rabbit anti-N-cadherin (green). Scale bar= 50 µm.


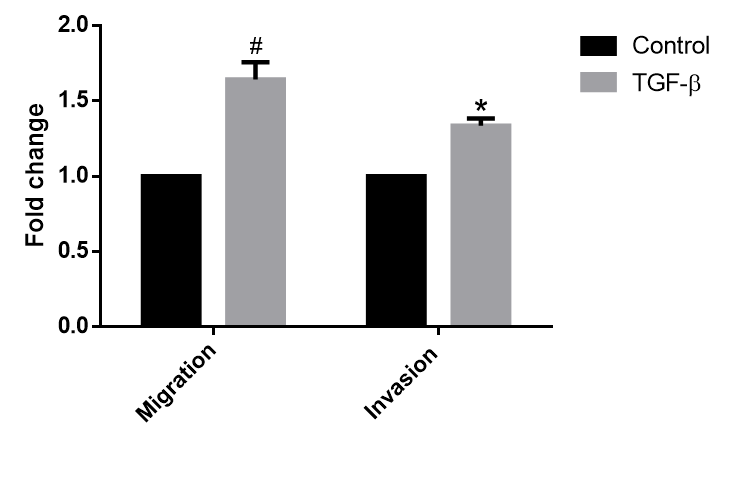


**Supplementary Fig. 5** Effects of BCDPs on A549 migration (A) and invasion (B) after 72 h of exposure to TGF-β (5 ng/mL). Each bar shows mean ± SEM of three independent experiments (N=3). Statistical analysis was performed by Two-way ANOVA with Sidak's multiple comparisons test. ^#^*p*<0.001 and **p*<0.05 vs control cells.
