# Supplementary material for: Combustion-derived particles from biomass sources differently promote epithelial-to-mesenchymal transition on A549 cells
Source: Arch Toxicol. 2021 Jan 22;95(4):1379–90. doi: 10.1007/s00204-021-02983-8 (PMC8032642; doi:10.1007/s00204-021-02983-8)
Supplement: Supplementary file 2 — Supplementary file2 (DOCX 1978 KB) [file 204_2021_2983_MOESM2_ESM.docx]

**Supplemental file 2**

**Archives of Toxicology**

**Combustion-derived particles from biomass sources differently promote epithelial to mesenchymal transition on A549 cells**

Sara Marchetti^1*^, Rossella Bengalli^1^, Pamela Floris^1^, Anita Colombo^1^, Paride Mantecca^1^

^1^POLARIS Research Centre, Department of Earth and Environmental Sciences, University of Milano-Bicocca, Piazza della Scienza 1, 20126 Milano, Italy

Sara Marchetti: ^1^POLARIS Research Centre, Department of Earth and Environmental Sciences, University of Milano-Bicocca, Piazza della Scienza 1, 20126 Milano, Italy. s.marchetti16@campus.unimib.it

tel: +390264482928

https://orcid.org/0000-0002-8767-9962

* Corresponding author


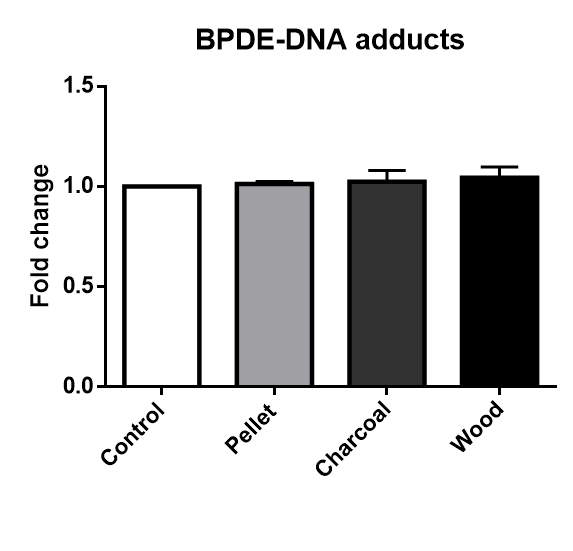


**Supplementary Fig. 1** BPDE adduct formation in A549 cells after 72 h of exposure to 2.5 µg/cm^2^. Each bar shows mean ± SEM of three independent experiments (N=3). Statistical analysis was performed by One-way ANOVA with Dunnett's multiple comparisons test.


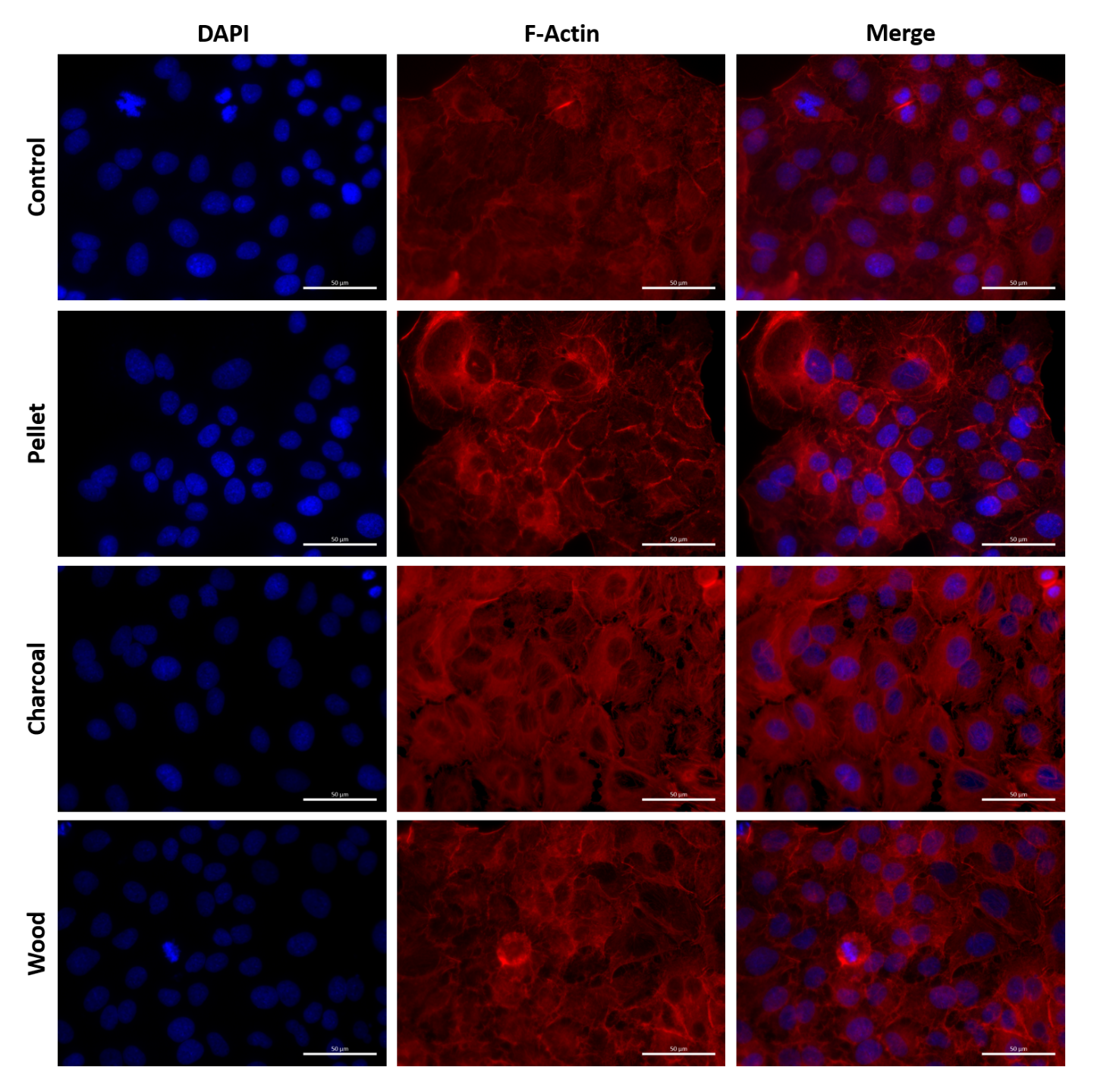


**Supplementary Fig. 2** Immunofluorescence on A549 cells after 72 h of exposure to BCDPs (2.5 µg/cm^2^). Nuclei are stained with DAPI (blue) and F-actin with rhodamine-phalloidin (red). Scale bar= 50 µm.
